# Editorial: Biosignal processing and computational methods to enhance sensory motor neuroprosthetics

**DOI:** 10.3389/fnins.2015.00434

**Published:** 2015-11-05

**Authors:** Mitsuhiro Hayashibe, David Guiraud, Jose L. Pons, Dario Farina

**Affiliations:** ^1^French Institute for Research in Computer Science and Automation, University of MontpellierMontpellier, France; ^2^Spanish National Research CouncilMadrid, Spain; ^3^Department of Neurorehabilitation Engineering, University Medical Center Göttingen, Georg-August UniversityGöttingen, Germany

**Keywords:** neuroprosthetics, electromyography, electroencephalography, brain-computer interface, neurorehabilitation

Neuroprosthetics is an interdisciplinary field of study that comprises neuroscience, computer science, physiology, and biomedical engineering. Each of these areas contributes to finally enhance the functionality of neural prostheses for the substitution or restoration of motor, sensory or cognitive funtions that might have been damaged as a result of an injury or a disease. For example, heart pace makers and cochlear implants substitute the functions performed by the heart and the ear by emulating biosignals with artificial pulses. These approaches require reliable bio-signal processing and computational methods to provide functional augmentation of damaged senses and actions.

This Research Topic aims at bringing together recent advances in sensory motor neuroprosthetics. This issue includes research articles in all relevant areas of neuroprosthetics: (1) biosignal processing, especially of Electromyography (EMG) and Electroencephalography (EEG) signals, and other modalities of biofeedback information, (2) computational methods for modeling parts of the sensorimotor system, (3) control strategies for delivering the optimal therapy, (4) therapeutic systems aiming at providing solutions for specific pathological motor disorders, (5) man-machine interfaces, such as a brain-computer interface (BCI), as an interaction modality between the patient and the neuroprostheses.

One challenging issue in motor prosthetics is the variability in the clinical presentation of patients, who show a variety of neurological disorders and physiological conditions. In order to improve neuroprosthetic performance beyond the current limited use, reliable bio-signal processing for extracting the intended neural information is needed (Farina et al., 2014). This information extraction stage can also be based on a modeling approach. Personalized neuroprosthetics with bio-signal feedback (Hayashibe et al., 2011; Borton et al., 2013; Li et al., 2014) could be a break-through toward intelligent neuroprosthetics. Combining different engineering techniques, such as in a hybrid approach (Del-Ama et al., 2014), is essential to expand the range of technological applications for wider patient populations. Recent advances of BCI are also relevant in this field to enable patients to transmit their intention of movement and its usage both for functional and rehabilitative purposes.

This Research Topic comprises original research activities in different levels of maturity ranging from hypothesis and poof-of-concept (Dutta et al., 2014; Grahn et al., 2014b) to systems already tested with some patients. It also contains a variety of approaches from computational method to experimental studies. Following the recent intensive developments of advanced BCI systems (Leeb et al., 2015; Muller-Putz et al., 2015), many contributions in this Research Topic are provided in the field of BCI, both with the aim of functional replacement and for neurorehabilitation. We overview those contributions for each category.

## 1. Signal processing of EMG and mechanical sensors

Cervical spinal cord injury (SCI) paralyzes muscles of the hand and arm, making it difficult to perform activities of daily living. Any reaching system requires a user interface to decode parameters of an intended reach. Corbett et al. (2014) present the benefits of combining different signal sources to control the reach in people with a range of impairments. A multimodal-decoding algorithm was developed while shoulder EMGs and gaze information were utilized for effective reaching task with assistive robot control, which provides guiding mobilization of the limb.

Powered prostheses are often controlled using EMG signals, which may introduce high levels of uncertainty even for simple tasks. According to Bayesian theories, higher uncertainty should influence how the brain adapts the motor commands in response to the perceived errors. Johnson et al. (2014) provide a simplified comparison framework of prosthesis and able-bodied control by studying adaptation with three control interfaces: joint angle, joint torque, and EMG. Increased errors and decreased visual uncertainty led to faster adaptation. This result suggests that Bayesian models are useful for describing prosthesis control and the man-machine interaction problem.

Lambrecht et al. (2014) present the first steps toward a more user-friendly and context-aware neuroprosthesis for tremor suppression and real-time monitoring. This methodology will enable the monitoring of tremor with context awareness by facilitating the automatic identification of the relative orientation of the sensor location.

## 2. Computational methods for modeling targeted sensori motor system and control of neuroprosthetics

This section overviews articles that are oriented toward new types of modeling and control for sensory motor neuroprosthetics.

An equilibrium-point control of human elbow-joint movement is proposed in Matsui et al. (2014) by using multichannel functional electrical stimulation. In this study, a computational electrical stimulation method that stimulates units of agonist-antagonist muscle pairs is developed. Muscle co-contraction level along with the total force was controlled for elbow joints with FES. In Klauer et al. (2014), a feedback control system is proposed for Neuro-Muscular Electrical Stimulation (NMES) to enable reaching in people with no residual voluntary control of the arm and shoulder due to high level SCI. NMES is applied to the deltoids and the biceps muscles and integrated with a three degrees of freedom (DoFs) passive exoskeleton, which partially compensates gravitational forces.

As for sensory modeling, Williams and Constandinou (2014) aimed at combining efficient implementations of biomechanical and proprioceptor models in order to generate signals that mimic human muscular proprioceptive patterns for future experimental work in prosthesis feedback. A neuro-musculoskeletal model of the upper limb with seven DoFs and 17 muscles is presented and generates real time estimates of muscle spindle and Golgi Tendon Organ neural firing patterns. The paper (Alnajjar et al., 2015) addresses the concept of sensory synergies. In contrast to muscle synergies, it hypothesizes that sensory synergies play an essential role in integrating the overall environmental inputs to provide low-dimensional information to the CNS. To examine the hypothesis, posture control experiments were conducted involving lateral disturbance on healthy participants.

Decoding the motor intent from recorded neural signals is essential for the development of neuroprostheses. To facilitate online decoding, Abdelghani et al. (2014) describe a software platform to simulate neural motor signals recorded with peripheral nerve electrodes, such as longitudinal intrafascicular electrodes (LIFEs). The simulator uses stored motor intent signals to drive a pool of simulated motoneurons with various spike shapes, recruitment characteristics, and firing frequencies.

A review article of Grahn et al. (2014a) summarizes neuroprosthetic technology for improving functional restoration following SCI and describes BCIs suitable for control of neuroprosthetic systems with multiple degrees of freedom. Additionally, stimulation paradigms that can improve synergy with higher planning centers and improve fatigue-resistant activation of paralyzed muscles are discussed.

## 3. Therapeutic systems targeted to specific pathological motor disorders

In this section, we overview the clinical applications enhanced by advanced computations.

Ortiz-Catalan et al. (2014) address the treatment of phantom limb pain (PLP) based on augmented reality and gaming controlled by myoelectric pattern recognition. The technology applied is non-invasive and combines the prediction of motion intent through the decoding of myoelectric signals, with the inclusion of virtual and augmented reality. As opposed to conventional mirror therapy, this system allows full range of motion and direct volitional control of the virtual limb.

Grahn et al. (2014b) demonstrate a neurochemical closed-loop controller for deep brain stimulation (DBS). This technology report article summarizes the current understanding of electrophysiological and electrochemical processing for control of neuromodulation therapies. Additionally, it describes a proof-of-principle closed-loop controller that characterizes DBS-evoked dopamine changes to adjust stimulation parameters in a rodent model of DBS.

Dutta et al. (2014) summarize post-stroke balance rehabilitation under multi-level electrotherapy. This hypothesis article presents a multi-level electrotherapy paradigm toward motor rehabilitation that postulates that while the brain acts as a controller to drive NMES, the state of the brain can be altered toward improvement of visuomotor task performance with non-invasive brain stimulation (NIBS). This leads to a multi-level electrotherapy paradigm where a virtual reality-based adaptive response technology is proposed for post-stroke balance rehabilitation.

## 4. BCI applied for neuroprosthetics enhancement

Here, we overview four articles related to motor intention extraction through brain signals for reaching and sit-standing by different approaches toward BCI-driven neuroprosthetics.

Choi (2013) presents the reconstruction of the joint angles of the shoulder and elbow from non-invasive electroencephalographic signals. The cortical activities were estimated from 64 channels electroencephalography (EEG) signals using the Hierarchical Bayesian estimation while continuous arm reaching movements. From the estimated cortical activities, a sparse linear regression method was used to reconstruct the electromyography (EMG) signals of nine arm muscles. Then, a modular artificial neural network was used to estimate four joint angles from the estimated EMG signals.

Morishita et al. (2014) address BMI to control a prosthetic arm with monkey's electrocorticography (ECoG) during periodic movements. This study demonstrated an improvement of the response time for detecting the motor intention from the cortical signal. It focused on the generation of a trigger event by decoding muscle activity in order to predict integrated electromyograms (iEMGs) from the ECoGs.

In Lew et al. (2014), single trial prediction of self-paced reaching directions from EEG signals is demonstrated. The feasibility of predicting movement directions in self-paced upper limb center-out reaching tasks in single trials is studied. Spontaneous movements executed without an external cue, are natural motor behavior in humans. Thus, BCI for self-paced motions is important. It reports results of non-invasive EEG recorded from mild stroke patients and healthy participants.

Bulea et al. (2014) discuss sitting and standing intention decoded from scalp EEG recorded prior to movement execution. Low frequency signals recorded from non-invasive EEG, in particular movement-related cortical potentials (MRPs), are associated with preparation and execution of the movement. The paper investigated the ability to decode movement intent from the delta-band (0.1–4 Hz) of the EEG signal recorded immediately before the movement execution in healthy volunteers. This study demonstrates that delta-band EEG recorded immediately before the movement carries discriminative information regarding movement type.

The detection of movement-related components is useful in brain-machine interfaces. A common approach is to classify the brain activity into a number of templates or states. However, complex arm movements such as reaching and grasping are prone to cross-trial variability due to the way movements are performed. The paper by Talakoub et al. (2015) presents a method of alignment that accounts for the variabilities in the way the movements are conducted. Arm speed was used to align neural activity. Four subjects had ECoG electrodes implanted over their primary motor cortex using the upper limb contralateral to the site of electrode implantation.

Human learning effect through neuro feedback in BCI are addressed in two articles in this Research Topic. In Prins et al. (2014), an adaptive BMI that can handle inaccuracies in the feedback is described and it is shown that it produces adaptive reinforcement learning based BMIs in a simulation study. A critic confidence measure, which indicated how appropriate the feedback is for updating the decoding parameters of the user is introduced. The results show that with the new update formulation, the critic accuracy is no longer a limiting factor for the overall performance.

Restorative BCI are increasingly used to provide feedback of neuronal states to normalize pathological brain activity and achieve behavioral gains. However, patients often show a large variability, or even inability of BCI control. The paper by Bauer and Gharabaghi (2015) presents a Bayesian model of neurofeedback and reinforcement learning for different threshold selection strategies in a simulation to study the impact of threshold adaptation of a linear classifier on optimizing restorative BCIs.

The contributions in this Research Topic describe a large variety of computational methods with unique approaches. As we have seen the necessity of different approaches for different applications, there are significant needs to correspond to patient-specific problems in neurorehabilitation and neuroprosthetics. This issue demonstrated a way to manage such complex scientific questions through biosignal processing and computational methods. The relevance of the presented contributions is testified by the fact that this Research Topic is the most viewed among all special issues in the category of neuroprosthetics under Frontiers in Neuroscience (61,182 views as of 20 Oct 2015). We would like to acknowledge all the authors of the 19 papers in this issue. As neurofeedback loop is essential to improve neuroprosthetic control, the exchanges and discussions in this interdisciplinary field will lead the advancement of neuroprosthetics technology with active information loop in our society. We hope this Research Topic may take a role of triggering synergistic effect for further development among researchers in this field.

### Conflict of interest statement

The authors declare that the research was conducted in the absence of any commercial or financial relationships that could be construed as a potential conflict of interest.

## References

[B1] Abdelghani M. N. Abbas J. J. Horch K. W. Jung R. (2014). A functional model and simulation of spinal motor pools and intrafascicular recordings of motoneuron activity in peripheral nerve. Front. Neurosci. 8:371. 10.3389/fnins.2014.00371 25452711 PMC4231878

[B2] Alnajjar F. Itkonen M. Berenz V. Tournier M. Nagai C. Shimoda S. (2015). Sensory synergy as environmental input integration. Front. Neurosci. 8:436. 10.3389/fnins.2014.00436 25628523 PMC4292368

[B3] Bauer R. Gharabaghi A. (2015). Reinforcement learning for adaptive threshold control of restorative brain-computer interfaces: a Bayesian simulation. Front. Neurosci. 9:36. 10.3389/fnins.2015.00036 25729347 PMC4325901

[B4] Borton D. Micera S. Millán J. D. R. Courtine G. (2013). Personalized neuroprosthetics. Sci. Transl. Med. 5, 210rv2. 10.1126/scitranslmed.3005968 24197737

[B5] Bulea T. C. Prasad S. Kilicarslan A. Contreras-Vidal J. L. (2014). Sitting and standing intention Can be decoded from scalp EEG recorded prior to movement execution. Front. Neurosci. 8:376. 10.3389/fnins.2014.00376 25505377 PMC4243562

[B6] Choi K. (2013). Reconstructing for joint angles on the shoulder and elbow from non-invasive electroencephalographic signals through electromyography. Front. Neurosci. 7:190. 10.3389/fnins.2013.00190 24167469 PMC3807039

[B7] Corbett E. A. Sachs N. A. Körding K. P. Perreault E. J. (2014). Multimodal decoding and congruent sensory information enhance reaching performance in subjects with cervical spinal cord injury. Front. Neurosci. 8:123. 10.3389/fnins.2014.00123 24904265 PMC4033069

[B8] del-Ama A. J. Gil-Agudo A. Pons J. L. Moreno J. C. (2014). Hybrid FES-robot cooperative control of ambulatory gait rehabilitation exoskeleton. J. Neuroeng. Rehabil. 11:27. 10.1186/1743-0003-11-27 24594302 PMC3995973

[B9] Dutta A. Lahiri U. Das A. Nitsche M. A. Guiraud D. (2014). Post-stroke balance rehabilitation under multi-level electrotherapy: a conceptual review. Front. Neurosci. 8:403. 10.3389/fnins.2014.00403 25565937 PMC4266025

[B10] Farina D. Jiang N. Rehbaum H. Holobar A. Graimann B. Dietl H.. (2014). The extraction of neural information from the surface EMG for the control of upper-limb prostheses: emerging avenues and challenges. IEEE Trans. Neural Syst. Rehabil. Eng. 22, 797–809. 10.1109/TNSRE.2014.2305111 24760934

[B11] Grahn P. J. Mallory G. W. Berry B. M. Hachmann J. T. Lobel D. A. Lujan J. L. (2014a). Restoration of motor function following spinal cord injury via optimal control of intraspinal microstimulation: toward a next generation closed-loop neural prosthesis. Front. Neurosci. 8:296. 10.3389/fnins.2014.00296 25278830 PMC4166363

[B12] Grahn P. J. Mallory G. W. Khurram O. U. Berry B. M. Hachmann J. T. Bieber A. J.. (2014b). A neurochemical closed-loop controller for deep brain stimulation: toward individualized smart neuromodulation therapies. Front. Neurosci. 8:169. 10.3389/fnins.2014.00169 25009455 PMC4070176

[B13] Hayashibe M. Zhang Q. Guiraud D. Fattal C. (2011). Evoked EMG-based torque prediction under muscle fatigue in implanted neural stimulation. J. Neural Eng. 8:064001. 10.1088/1741-2560/8/6/064001 21975831

[B14] Johnson R. E. Kording K. P. Hargrove L. Sensinger J. W. (2014). Does EMG control lead to distinct motor adaptation? Front. Neurosci. 8:302. 10.3389/fnins.2014.00302 25324712 PMC4179747

[B15] Klauer C. Schauer T. Reichenfelser W. Karner J. Zwicker S. Gandolla M.. (2014). Feedback Control of arm movements using neuro-muscular electrical stimulation (NMES) combined with a lockable, passive exoskeleton for gravity compensation. Front. Neurosci. 8:262. 10.3389/fnins.2014.00262 25228853 PMC4151235

[B16] Lambrecht S. Gallego J. A. Rocon E. Pons J. L. (2014). Automatic real-time monitoring and assessment of tremor parameters in the upper limb from orientation data. Front. Neurosci. 8:221. 10.3389/fnins.2014.00221 25120424 PMC4110507

[B17] Leeb R. Tonin L. Rohm M. Desideri L. Carlson T. Millan J. D. R. (2015). Towards independence: a BCI telepresence robot for people with severe motor disabilities. Proc. IEEE 103, 969–982. 10.1109/JPROC.2015.2419736

[B18] Lew E. Y. L. Chavarriaga R. Silvoni S. Millán J. D. R. (2014). Single trial prediction of self-paced reaching directions from EEG signals. Front. Neurosci. 8:222. 10.3389/fnins.2014.00222 25136290 PMC4117993

[B19] Li Z. Hayashibe M. Fattal C. Guiraud D. (2014). Muscle fatigue tracking with evoked EMG via recurrent neural network: toward personalized neuroprosthetics. Comput. Intell. Mag. IEEE 9, 38–46. 10.1109/MCI.2014.2307224

[B20] Matsui K. Hishii Y. Maegaki K. Yamashita Y. Uemura M. Hirai H.. (2014). Equilibrium-point control of human elbow-joint movement under isometric environment by using multichannel functional electrical stimulation. Front. Neurosci. 8:164. 10.3389/fnins.2014.00164 24987326 PMC4060571

[B21] Morishita S. Sato K. Watanabe H. Nishimura Y. Isa T. Kato R.. (2014). Brain-machine interface to control a prosthetic arm with monkey ECoGs during periodic movements. Front. Neurosci. 8:417. 10.3389/fnins.2014.00417 25565947 PMC4264470

[B22] Muller-Putz G. Leeb R. Tangermann M. Hohne J. Kubler A. Cincotti F. (2015). Towards noninvasive hybrid brain–computer interfaces: framework, practice, clinical application, and beyond. Proc. IEEE 103, 926–943. 10.1109/JPROC.2015.2411333

[B23] Ortiz-Catalan M. Sander N. Kristoffersen M. B. Hå kansson B. Brå nemark R. (2014). Treatment of phantom limb pain (PLP) based on augmented reality and gaming controlled by myoelectric pattern recognition: a case study of a chronic PLP patient. Front. Neurosci. 8:24. 10.3389/fnins.2014.00024 24616655 PMC3935120

[B24] Prins N. W. Sanchez J. C. Prasad A. (2014). A confidence metric for using neurobiological feedback in actor-critic reinforcement learning based brain-machine interfaces. Front. Neurosci. 8:111. 10.3389/fnins.2014.00111 24904257 PMC4033619

[B25] Talakoub O. Popovic M. Navarro J. Hamani C. Fonoff E. Wong W. (2015). Temporal alignment of electrocorticographic recordings for upper limb movement. Front. Neurosci. 8:431. 10.3389/fnins.2014.00431 25628522 PMC4292555

[B26] Williams I. Constandinou T. G. (2014). Computationally efficient modeling of proprioceptive signals in the upper limb for prostheses: a simulation study. Front. Neurosci. 8:181. 10.3389/fnins.2014.00181 25009463 PMC4069835

